# Contributions of Animal Models to the Mechanisms and Therapies of Transthyretin Amyloidosis

**DOI:** 10.3389/fphys.2019.00338

**Published:** 2019-04-02

**Authors:** Ridwan Babatunde Ibrahim, Yo-Tsen Liu, Ssu-Yu Yeh, Jin-Wu Tsai

**Affiliations:** ^1^Institute of Brain Science, School of Medicine, National Yang-Ming University, Taipei, Taiwan; ^2^Taiwan International Graduate Program in Interdisciplinary Neuroscience, National Yang-Ming University and Academia Sinica, Taipei, Taiwan; ^3^Department of Neurology, Neurological Institute, Taipei Veterans General Hospital, Taipei, Taiwan; ^4^Department of Medicine, School of Medicine, National Yang-Ming University, Taipei, Taiwan; ^5^Brain Research Center and Biophotonics and Molecular Imaging Research Center, National Yang-Ming University, Taipei, Taiwan

**Keywords:** transthyretin, familial amyloid polyneuropathy, *C. elegans*, *D. melanogaster*, transgenic mouse, iPSC, neurodegenerative disorder, animal model

## Abstract

Transthyretin amyloidosis (ATTR amyloidosis) is a fatal systemic disease caused by amyloid deposits of misfolded transthyretin, leading to familial amyloid polyneuropathy and/or cardiomyopathy, or a rare oculoleptomeningeal amyloidosis. A good model system that mimic the disease phenotype is crucial for the development of drugs and treatments for this devastating degenerative disorder. The present models using fruit flies, worms, rodents, non-human primates and induced pluripotent stem cells have helped researchers understand important disease-related mechanisms and test potential therapeutic options. However, the challenge of creating an ideal model still looms, for these models did not recapitulates all symptoms, particularly neurological presentation, of ATTR amyloidosis. Recently, knock-in techniques was used to generate two humanized ATTR mouse models, leading to amyloid deposition in the nerves and neuropathic manifestation in these models. This review gives a recent update on the milestone, progress, and challenges in developing different models for ATTR amyloidosis research.

## Introduction

Transthyretin amyloidosis (ATTR) is the most common type of autosomal-dominant hereditary systemic amyloidosis associated with the transthyretin (TTR) protein; with either early- or late-onset presentation in patients (Benson and Kincaid, 2007; Plante-Bordeneuve and Said, 2011; Adams et al., 2012). ATTR can arise either due to senescent event associated with the wild-type *TTR* gene leading to wild-type amyloidosis (formerly known as senile systemic amyloidosis), or hereditary ATTR due to mutations in the *TTR* gene, leading to either familial amyloid polyneuropathy (ATTR-FAP), familial amyloid cardiomyopathy (ATTR-FAC), or a rare form of oculoleptomeningeal amyloidosis (OLMA) (Ando et al., 2005; Rapezzi et al., 2010; Leung et al., 2013; Mathieu et al., 2018).

TTR is a secreted homotetrameric protein found largely in the plasma and cerebrospinal fluid (CSF) (Almeida et al., 2004; Buxbaum and Reixach, 2009). It is primarily synthesized in the liver, which provides most plasma pool of the protein (Ingenbleek and Bernstein, 2015), and in the choroid plexus of the brain contributing to its presence in the CSF (Richardson et al., 2015; Johnson et al., 2018). TTR is also synthesized in the retinal pigment epithelium (RPE) of the eye (Pfeffer et al., 2004). Its main function is to transport thyroid hormone and retinol throughout the body (Buxbaum and Reixach, 2009; Prapunpoj and Leelawatwattana, 2009). In ATTR amyloidosis, deposition of unfolded TTR aggregates are found in the heart, peripheral nerves and other organs, leading to multiple dysfunctions of these organs (Buxbaum and Reixach, 2009).

## Pathophysiology and Clinical Features of Attr Amyloidosis

Following its description by Andrade in 1952, the number of identified causative mutations in *TTR* for transthyretin amyloidosis has increased (Benson, 2017). Currently there are over 100 mutations in the *TTR* gene known to cause the disease, most being single amino acid substitution (Benson, 2017). The most common ATTR mutation is a substitution of valine residue for methionine at position 30 (p.Val30Met) (Stewart et al., 2018). This mutation alters the native tetrameric structure of circulating TTR, promoting release of amyloidogenic TTR monomers which misfold and deposit as amyloid at target organs (Ando et al., 2005; Coelho et al., 2016). Most people affected with hereditary ATTR amyloidosis are heterozygous, expressing both normal and variant TTR protein (Hamilton and Benson, 2001; Suhr et al., 2003).

**FIGURE 1:**
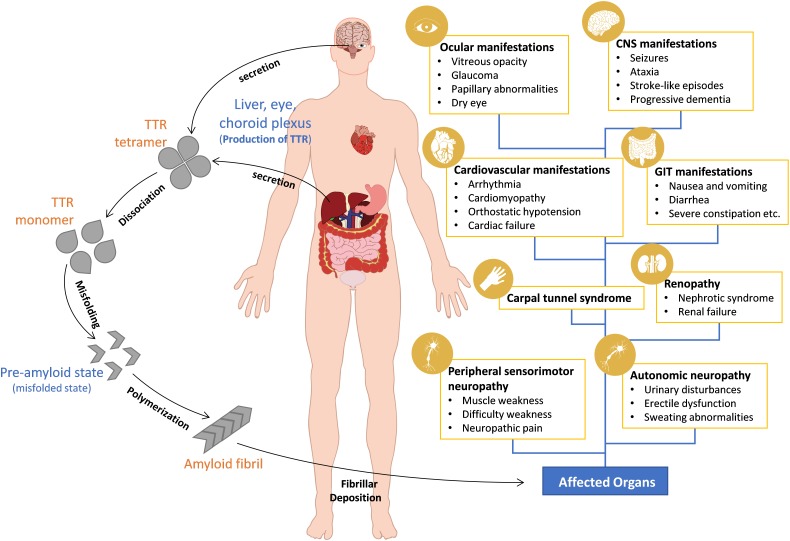
Pathogenesis and clinical manifestations of ATTR amyloidosis. Wild-type and mutant TTR are produced and secreted into the blood or cerebrospinal fluid (CSF) as tetrameric proteins. Mutations in the TTR gene makes the tetrameric TTR protein dissociate into aggregate prone monomers that misfold and aggregate into amyloid fibrils which deposit in the heart, peripheral nerves, and other organs. Clinical manifestations depend mainly on the site of TTR amyloid deposition.

The clinical phenotype of variant TTR amyloidosis varies greatly by its mutation, age of onset, disease penetrance and prognosis (Ruberg and Berk, 2012). Clinically, ATTR-FAP patients manifest irreversible progressive sensorimotor and autonomic neuropathic symptoms (Hund, 2012). Symptoms start with discomfort in the feet, spontaneous pain and numbness moving proximally to the thigh and then to the upper limbs (Plante-Bordeneuve and Said, 2011; Hund, 2012). Patient deaths are inevitable around 11 years from disease onset (Koike et al., 2012; Adams, 2013). In ATTR-FAC patients, symptoms like diastolic and systolic dysfunction, arrhythmias and other signs of progressive heart failure are common (Castano et al., 2015). In some cases, patients have both peripheral neuropathy and cardiac involvement (Haagsma et al., 2004). In rare cases of OLMA, seizures, ataxia, vitreous opacity, and glaucoma are common symptoms (McColgan et al., 2015; Mathieu et al., 2018) (Figure 1).

## Current and Emerging Therapies for Attr Amyloidosis

Currently, treatment options for ATTR amyloidosis are limited but liver transplantation (LT) serve as an established option for early-stage disease patients (Gertz, 2017). LT has been shown to improve patient’s survival as it replaces production of mutant TTR with wild-type TTR proteins via the donor organ (Carvalho et al., 2015; Ericzon et al., 2015). However, several limitations to the use of LT have been reported, including toxicities associated with immunosuppression, incidence of hepatic artery thrombosis etc. (Carvalho et al., 2015; Hawkins et al., 2015; Adams et al., 2017) expediting the need for alternative therapies for ATTR amyloidosis. Multiple potential therapies targeting the underlying mechanisms involved in ATTR amyloidosis are emerging (see Ueda and Ando, 2014; Gertz et al., 2015; Nuvolone and Merlini, 2017; Plante-Bordeneuve, 2018 for a review).

**FIGURE 2:**
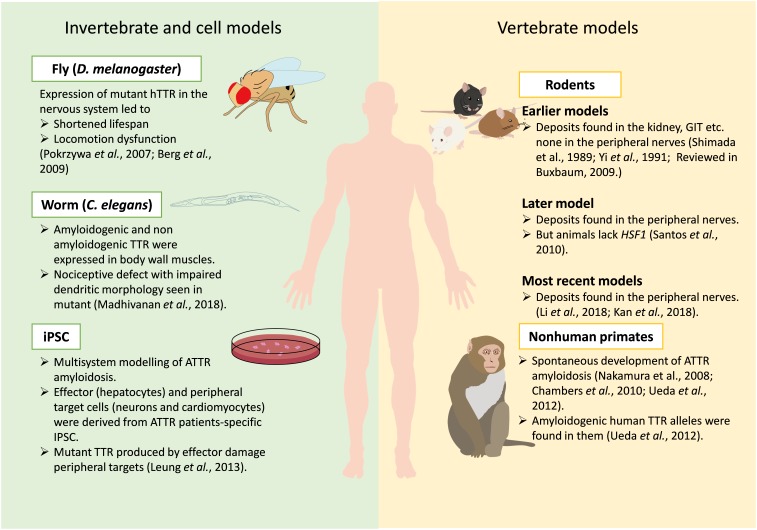
Current models available for studying ATTR amyloidosis. Various models for ATTR amyloidosis are depicted in this figure, including invertebrate, cell and vertebrate models. Key phenotypes and findings from these models are indicated with proper references.

Although creating an ideal animal model can be challenging, they play invaluable roles in our understanding of disease pathology and discovery of new drug targets and biomarkers (McGonigle and Ruggeri, 2014; Bolker, 2017). A lot of effort is being put into generating successful models for ATTR amyloidosis that can recapitulate all human characteristics of the disease. However, earlier models could not recapitulate all human pathology of ATTR amyloidosis (see Buxbaum, 2009 for a review). Recently, two models show deposition of TTR deposits in the nerves a major breakthrough in modeling ATTR amyloidosis in mice. This review aims to give an update on the evolution of animal and cell models studied in ATTR amyloidosis, from invertebrate animals, including worms (*Caenorhabditis elegans*) and flies (*Drosophila melanogaster*), to mice, non-human primates, and lastly induced pluripotent stem cells (iPSC). We surveyed literatures from PubMed using keywords “Animal models for TTR amyloidosis; Transthyretin FAP animal models; Transthyretin amyloidosis models.” The use of these models in testing potential therapeutic targets have also been highlighted (Figure 2).

## Invertebrate Models

The most widely used invertebrate model organisms for biological research, *D. melanogaster* and *C. elegans*, have been used to model ATTR amyloidosis. The existence of conserved molecular pathways between human and invertebrates (Lev et al., 1999; Plowman et al., 1999) coupled with the availability of great genetics, cellular and molecular biology tools makes them good systems for modeling brain diseases and drug discovery research (Alexander et al., 2014; Nussbaum-Krammer and Morimoto, 2014; Ugur et al., 2016).

Since expressing toxic human proteins in invertebrates may mimic some aspects of disease conditions (Muqit and Feany, 2002), the expression of TTR mutants in the fly led to phenotypes partially evocative of the human disease (Figure 2). Expressing ATTR V30M in the nervous system resulted in progressive age-dependent reduced climbing activity and lifespan while TTR WT showed milder phenotype (Berg et al., 2009). Positive amyloid staining by Congo red was also seen in aged ATTR V30M flies (Berg et al., 2009). Similar outcomes were observed with ATTR L55P and an engineered variant TTR-A (mutant with two amino acid replacement TTR V14N/V16E). They showed time-dependent aggregation of misfolded TTR, neurodegeneration, shortened lifespan, and compromised flying ability (Pokrzywa et al., 2007). Although these fly models reveal cell-autonomous toxicity of mutant *hTTR* instead of cell non-autonomous toxicity as seen in human, they have been used to test peptides which prevent TTR aggregation *in vitro* (Saelices et al., 2018).

To recapitulate cell non-autonomous toxicity, Madhivanan et al. (2018) created a *C. elegans* model for ATTR amyloidosis, which expresses human TTR in the body wall muscle. Wild-type TTR and mutants (T119M, V30M, and D18G) were expressed in the body-wall muscle using the *unc-54* promoter. Insoluble forms of TTR were detected in C. *elegans* expressing both ATTR V30M and ATTR D18G mutants (Figure 2). Modeling the differential secretory properties of WT-TTR and other mutants reveal absence of TTR D18G signals from the body cavity, an indication of ineffective secretion due to its highly unstable nature (Hammarstrom et al., 2003; Sekijima et al., 2005; Madhivanan et al., 2018). Expressing ATTR V30M in *C. elegans* led to nociception impairment analogous to human FAP patients. This also led to significant impairment on dendritic morphology of somatosensory neurons in ATTR V30M animals (Madhivanan et al., 2018). Overall modeling ATTR V30M in *C. elegans* led to cell non-autonomous neurotoxicity that has not been demonstrated in previous fly models.

## Rodent Attr Models

Attempts to model ATTR amyloidosis in mice began more than 3 decades ago by random genomic insertion of wild-type or mutant hTTR. However, efforts to produce an ideal mouse model for ATTR amyloidosis have not been as successful as scientists would desire (Buxbaum et al., 2003; Buxbaum, 2009; Kan et al., 2018; Li et al., 2018). To gain insights into the pathogenesis of ATTR amyloidosis, transgenic ATTR V30M mice were created with the mouse metallothionein promoter (Shimada et al., 1989). Despite appreciable serum levels of human TTR in these mice, no amyloid deposits were observed in their peripheral (PNS) and autonomic nervous system (ANS). An improved transgenic line with increased gene copies of TTR V30M was later established (Yi et al., 1991). Slight TTR amyloid deposits were found in the kidneys, gastrointestinal tract (GIT) and cardiovascular organs of these mice at 6 months (Figure 2). Despite extensive amyloid deposition resembling SSA-like amyloid deposits at 24 months, no amyloid deposit was detected in their choroid plexus, PNS and ANS (Yi et al., 1991; Hamilton and Benson, 2001; Buxbaum, 2009). Subsequently, several research groups have generated transgenic animals from wild-type and other mutant TTR gene constructs; none had congophilic deposits in the peripheral nerves (see Buxbaum, 2009 for a review).

The activation of inflammatory and oxidative stress pathways by TTR deposits in patients (Sousa et al., 2000, 2001) led to the hypothesis that disruption of stress response pathway may accelerate TTR deposition. When transgenic mice expressing human TTR V30M were crossed with mice lacking heat shock transcription factor (Hsf1), their offspring showed early and extensive deposition of non-fibrillar TTR in the GIT and subsequent fibrillar non-congophilic deposit in sciatic nerve and dorsal root ganglia for the first time (Santos et al., 2010). Although there are lots of unanswered question about this model due to the absence of Hsf1, making it less optimal for either fine analysis or experimental therapies (Buxbaum, 2009), it has been used in numerous studies to understand pathogenesis of ATTR amyloidogenesis and the preclinical evaluation of pharmacotherapy and gene therapy studies (Cardoso et al., 2010; Ferreira et al., 2014, 2016; Butler et al., 2016).

In hTTR V30M *Hsf1^+/-^* mice, TTR-targeting siRNA-mediated silencing of hepatic TTR expression inhibit TTR deposition and facilitate regression of existing TTR deposits in tissues (Butler et al., 2016). In addition, the TTR stabilizer, tafamidis led to regression of TTR deposits across a broad range in affected tissues, together with reducing serum TTR protein concentration in hTTR V30M *Hsf1^+/-^* mice. This led to the approval of Patisiran by the US FDA and Tafamidis by the Japanese and European drug agencies for the management of ATTR amyloidosis after clinical trials. Patisiran, an RNAi therapeutic, was documented to successfully improve multiple clinical manifestations of hereditary transthyretin amyloidosis (Adams et al., 2018). Tafamidis on the other hand effectively delay progression of TTR-related polyneuropathy and lower rates of cardiovascular-related hospitalizations in patients (Maurer et al., 2018; Mundayat et al., 2018).

Nevertheless, the search for an ideal mouse model isn’t over until a model which mimic ATTR amyloidosis patients in symptoms and tissue-distribution of congophilic TTR amyloid deposit is achieved. Two groups recently generated mouse models with amyloid deposits in the peripheral nerves without additional genetic changes such as Hsf1 deficiency (Kan et al., 2018; Li et al., 2018). Biophysical analysis alongside biochemical studies in mice reveal that murine TTR inhibit aggregation and deposition of highly unstable human TTR via formation of a highly stable human-mouse heterotetramers (Tagoe et al., 2007; Reixach et al., 2008). To overcome this, Li et al. (2018), produced humanized mouse strains at both the *Ttr* and *Rbp4* loci allowing human TTR (hTTR) to associate with human RBP4 (hRBP4). Two mouse lines; normal (Ttr^hTTRV 30/hTTRV 30^: Rbp4^hRBP4/hRBP4^, abbreviated as *hV/hV:hR/hR*) and mutant (Ttr^hTTRV 30/hTTRM30^:Rbp4^hRBP4/hRBP4^, abbreviated as *hV/hM:hR/hR*) were generated. Remarkably, even though both hTTR and hRBP4 were found at relatively low levels in the serum of these mouse lines, these mice present with distinct amyloid deposition than previous conventional transgenic models. Amyloid deposits was first noticed in the GIT of *hV/hV:hR/hR* and *hV/hM:hR/hR* mice at 12 and 18 months respectively increasingly with age. At 24 months, amyloid deposition was observed in the heart of both lines with more deposit in the mutant mice. Notably at 24 months, Congo red positive amyloid deposits were found in the perineurium of the sciatic nerve of the mutant line (Li et al., 2018). This study further reveal the impact RBP4 might have on amyloid deposition as it is known to prevent TTR amyloid formation at lower pH (White and Kelly, 2001). A limitation to these successful models might be their inability to symbolize human TTR status due to their low serum hTTR level and high proportion of unbound TTR with RBP4 as it is unknown whether amyloid deposition occur in human under such low serum level of hTTR.

Of the earliest signs of ATTR-FAP is gradual degeneration of sensory nerve cells leading to abnormal sensations such as pain (Plante-Bordeneuve and Said, 2011; Ando et al., 2013). Although previous ATTR-FAP mutant mice have helped researchers gain insights into the pathogenesis of the disease (Buxbaum, 2009; Li et al., 2018), none of them replicate ATTR-FAP’s early pain symptoms. A human TTR knock-in mice established by replacing one allele of the mouse *Ttr* locus with human TTR A97S (hTTR^A97S^) recapitulate these symptoms (Kan et al., 2018). The TTR-A97S mutation is a hotspot Taiwanese mutation characterized by late onset presentation in patients marked with axonal degeneration (Liu et al., 2008; Yang et al., 2010; Chao et al., 2015). To achieve this, knock-in mice for both wild-type and ATTR A97S were generated by replacing m*Ttr* with hTTR via homologous recombination without altering the promoter and enhancer sequences of the m*Ttr*. The knock-in mice were backcrossed to C57BL/6 females to obtain heterozygotes with genotypes h*TTR*^wt^/m*Ttr*^wt^ (abbreviated as hTTR^wt^) and h*TTR*^A97S^/m*Ttr*^wt^ (abbreviated as hTTR^A97S^) (Kan et al., 2018). Serum and liver hTTR protein levels were detectable in both mice with lower levels in hTTR^A97S^ animals. Congophilic amyloid deposits were detected in the epineurium of the sural nerve, distal convoluted tubule of the kidney and other organs in aging hTTR^A97S^ (>2 years) mice but not in the adult group (8–56 weeks). Aging hTTR^A97S^ mice also exhibit hypersensitivity to mechanical stimuli, less nerve fiber density and less nerve sensation which are early manifestations of ATTR-FAP (Kan et al., 2018). Despite lack of motor deficit in the hTTR^A97S^ mouse, the presence of congophilic deposits plus display of early ATTR-FAP symptoms makes it a good model to test drugs targeting early-stage symptoms of ATTR-FAP.

## Non-Human Primate Models

Spontaneous development of ATTR amyloidosis have been reported in several vervet monkeys (Nakamura et al., 2008; Chambers et al., 2010; Ueda et al., 2012). In the monkeys observed by Ueda et al. (2012) aged vervets were observed to have cardiac arrhythmia, bradycardia and reduced ejection fraction in the heart mimicking human ATTR WT amyloidosis. Sequencing data reveal the presence of the amyloidogenic human TTR Ile 122 allele in these vervets, suggesting its pathogenic role in their development of ATTR amyloidosis. Other human amyloidogenic TTR alleles were also documented in non-human primates: Thr81 in New World monkeys (*Callithrix jacchus*) and the black-bearded saki (*Chiropotes satanas*) and Val107 in all New World monkeys (Ueda et al., 2012). These evidences are suggestive of non-human primates as possible valid pathologic models for ATTR amyloidosis.

## iPsc Models of Attr Amyloidosis

George J. Murphy’s group generated the first known patient-specific induced pluripotent stem cell (iPSC) library for ATTR amyloidosis (Leung and Murphy, 2016; Giadone et al., 2018). This development may help resolve existing variations between patients with identical TTR mutations in readiness for personalized ATTR amyloidosis modeling. IPSC lines from a ATTR L55P patient was generated and subsequently differentiated into hepatocytes (which produce mutant L55P TTR) and peripheral targets (cardiomyocytes and neurons) for ATTR amyloid deposition (Leung et al., 2013). IPSC derived hepatocytes successfully secrete TTRs which were internalized by IPSC neuronal-lineage cells as previously reported (Fleming et al., 2009). Stress-response markers and other known associated genes were upregulated in peripheral targets exposed to ATTR ^L55P^ media (Sousa et al., 2005; Leung et al., 2013). This successful patient derived IPSCs could be helpful in understanding complex etiology of the disease.

## Conclusion and Perspective

A good animal model is one which can replicate the human disease by depicting key pathologic and physiologic symptoms. Such model would satisfy the laid criteria for animal validation (Belzung and Lemoine, 2011). Despite recent success in the generation of mouse models with amyloid deposits in the nerves (Kan et al., 2018; Li et al., 2018), discrepancies in key physiologic and behavioral features of ATTR amyloidosis were seen. For example, amyloid deposits were seen earlier in mice carrying the normal human TTR gene (Li et al., 2018), a phenomenon inconsistent with human patient (Ton et al., 2014). Lack of motor deficit was also seen in the model by Kan et al., 2018. Slight modifications of these models may be needed to rectify these discrepancies. Considering the wide genotypic and phenotypic spectrum of this disease, generating mutant-specific ATTR animal models may be required to address specific questions that might allow construction of mutant-specific hypotheses essential to making further breakthroughs.

However, by combining different models, it might be possible to eventually replicate the clinical condition better. The challenge is to identify which aspect of the human disease a particular model addresses. Using these models, we have learnt important pathogenic mechanisms associated with ATTR amyloidosis, including aging-related oxidation, histological inflammation, thermodynamic stability, etc. Based on this knowledge, there have been several mechanism-driven therapeutic targets identified leading to the approval of Patisiran and Tafamidis for the management of this disease. Although this current therapies have achieved significant progress in certain indices for both peripheral neuropathy and cardiopathy, a multi-target drug design may be required to adequately manage the disease.

## Author Contributions

J-WT finalized the manuscript. All authors prepared the manuscript.

## Conflict of Interest Statement

The authors declare that the research was conducted in the absence of any commercial or financial relationships that could be construed as a potential conflict of interest.
